# Passive elongation of muscle fascicles in human muscles with short and long tendons

**DOI:** 10.14814/phy2.13528

**Published:** 2017-11-30

**Authors:** Jeanette M. Thom, Joanna Diong, Peter W. Stubbs, Robert D. Herbert

**Affiliations:** ^1^ School of Medical Sciences UNSW Sydney Sydney Australia; ^2^ Neuroscience Research Australia (NeuRA) Sydney Australia; ^3^ Sydney Medical School University of Sydney Sydney Australia; ^4^ Hammel Neurorehabilitation and Research Centre Aarhus University Aarhus Denmark

**Keywords:** Brachialis, fascicle length, gastrocnemius, tendon, tibialis anterior, ultrasonography

## Abstract

This study tested the hypothesis that the ratio of changes in muscle fascicle and tendon length that occurs with joint movement scales linearly with the ratio of the slack lengths of the muscle fascicles and tendons. We compared the contribution of muscle fascicles to passive muscle‐tendon lengthening in muscles with relatively short and long fascicles. Fifteen healthy adults participated in the study. The medial gastrocnemius, tibialis anterior, and brachialis muscle‐tendon units were passively lengthened by slowly rotating the ankle or elbow. Change in muscle fascicle length was measured with ultrasonography. Change in muscle‐tendon length was calculated from estimated muscle moment arms. Change in tendon length was calculated by subtracting change in fascicle length from change in muscle‐tendon length. The median (IQR) contribution of muscle fascicles to passive lengthening of the muscle‐tendon unit, measured as the ratio of the change in fascicle length to the change in muscle‐tendon unit length, was 0.39 (0.26–0.48) for the medial gastrocnemius, 0.51 (0.29–0.60) for tibialis anterior, and 0.65 (0.49–0.90) for brachialis. Brachialis muscle fascicles contributed to muscle‐tendon unit lengthening significantly more than medial gastrocnemius muscle fascicles, but less than would be expected if the fascicle contribution scaled linearly with the ratio of muscle fascicle and tendon slack lengths.

## Introduction

When relaxed muscle‐tendon units are passively lengthened, passive forces are generated in muscle fascicles and tendons. These forces elongate muscle fascicles and tendons, both of which contribute to the increases in length of the muscle‐tendon unit. Under passive conditions, change in muscle pennation contributes relatively little to increases in the length of the muscle‐tendon unit (Zajac 1989; Herbert et al. 2002, 2015; Hoang et al. 2007).

Tendons are less compliant than muscle fascicles. Nonetheless, in the relaxed human gastrocnemius and tibialis anterior muscles, tendons contribute more than muscle fascicles to passive changes in muscle‐tendon length (Herbert et al. 2002). When the gastrocnemius is passively lengthened, tendinous structures in series with the gastrocnemius muscle fascicles (the Achilles tendon, the tendinous aponeuroses, and the proximal gastrocnemius tendons) contribute between half and three‐quarters of the total change in muscle‐tendon length (Herbert et al. 2002, 2011, 2015; Hoang et al. 2007; Guilhem et al. 2016). Similarly, the tendinous structures in series with the tibialis anterior contribute nearly half of the of the total change in tibialis anterior muscle‐tendon length (Herbert et al. 2002). Both the gastrocnemius and tibialis anterior have tendons that are much longer than their muscle fascicles.

On theoretical grounds, it might be expected that tendons contribute more to muscle‐tendon lengthening in muscle‐tendon units which have relatively long tendons (Appendix 1). This study tests the hypothesis that the ratio of changes in muscle fascicle and tendon length scales linearly with the ratio of the slack lengths of the muscle fascicles and tendons.

## Materials and Methods

### Participants

Fifteen healthy, recreationally active adults participated in the study (7 males, 8 females; age 32 ± 9 years; height 1.69 ± 0.09 m; weight 67 ± 15 kg; data shown as means ± SD). Participants were recruited from the University community and had no musculoskeletal injuries, pathology or previous surgery at the muscles or joints concerned. All participants were given a participant information sheet prior to commencing the study and written informed consent was obtained. Approval for the study was obtained by the Human Research Ethics Committee [ref no: 12/076 (HREC/12/POWH/255)]. The study procedures conformed to the Helsinki Declaration on Human Experimentation.

### Procedures

Participants were seated on an isokinetic dynamometer (Cybex Norm with HUMAC, CSMi, Stoughton, MA, USA) and were tested either by passively rotating the ankle joint to obtain change in muscle fascicle lengths of the medial gastrocnemius and tibialis anterior muscles, or by passively rotating the elbow joint to obtain change in muscle fascicle lengths of the brachialis muscle. The joint was rotated through its full range of motion at 5°/sec (Herbert et al. 2015).

When testing the medial gastrocnemius and tibialis anterior muscles, participants were seated on the dynamometer with the left leg knee semiflexed at 68 ± 8° (mean ± SD). The left foot was placed on a footplate with the axis of rotation of the dynamometer aligned with the ankle axis of rotation and the dynamometer passively rotated the ankle into dorsiflexion and plantarflexion. Surface electrodes (diameter 30 mm, center‐center spacing ~35 mm) were placed on the mid portion of the lateral gastrocnemius and proximal portion of the tibialis anterior muscle bellies to monitor muscle activity (electromyography; EMG). The footplate was then positioned at 90° (neutral ankle angle) and two maximum voluntary contractions (MVC) each of plantarflexion and dorsiflexion were obtained.

When testing the brachialis muscle, participants were seated on the dynamometer with the left hand holding the lever attachment such that the axis of rotation of the dynamometer was aligned with the elbow axis of rotation. The elbow was free to rotate into flexion and extension; however, the range elbow flexion was limited because ultrasound transducers were placed over the arm to image brachialis. Surface electrodes were placed over the mid‐portion of the biceps brachii muscle belly to monitor EMG. The lever was then positioned at 90° elbow flexion and two elbow flexion MVCs were obtained.

**Figure 1:**
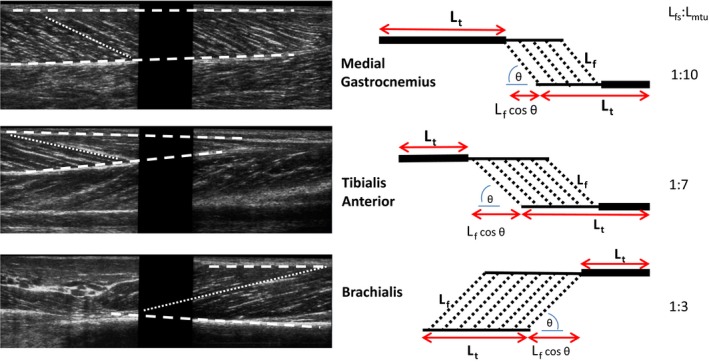
Ultrasound images of the medial gastrocnemius, tibialis anterior, and brachialis muscles (left) and schematic diagrams (right) showing how tendon lengths (*L*_t_), fascicle lengths (*L*_f_), and pennation angles (*θ*) were measured. The estimated ratio of fascicle slack length and muscle‐tendon slack length (*L*_fs_:*L*_mtus_) is given.

Ultrasound images of muscle fascicles were obtained with two ultrasound probes (Esaote MyLab25, 46 mm linear array probe LA522E operating at 12 Hz; Esaote, Genoa, Italy) held in series using a custom‐made mould (Herbert et al. 2015). The probes were placed over the mid‐point of the muscle belly and adjusted to obtain the clearest possible images in the plane of the muscle fascicles (Herbert et al. 2011, 2015; Diong et al. 2013) (Fig. 1). Images were judged to be in the approximate plane of the muscle fascicles when the proximal and distal ends of target fascicles could be visualized. The joint was first rotated through range at 5°/sec for at least three full flexion‐extension cycles to provide some control of history‐dependent muscle viscoelastic effects. Data were then recorded from the next three flexion‐extension cycles. Ultrasound images were digitally sampled at 10 Hz. Joint angle signals were sampled at 50 Hz. EMG signals were bandpass filtered at 10–500 Hz and sampled at 2000 Hz. Joint angle and EMG data were collected using Spike2 software and synchronized with the ultrasound images using the S2video plug‐in (Herbert et al. 2015) (CED 1401 interface, Cambridge, UK).

### Image analysis

For each muscle‐tendon unit, change in muscle fascicle length was measured from ultrasound images of the muscle fascicles. Trials in which there was detectable EMG (>5% of EMG at MVC) were excluded from the analyses. Of the remaining trials, the trial with the clearest image was analyzed. Procedures used to analyze ultrasound images have been described elsewhere (Herbert et al. 2011, 2015; Diong et al. 2013). Briefly, images from the two ultrasound transducers were cropped and stitched together offline to form a composite image (field of view of 110 mm, depth 40 mm, with an 18 mm gap in the middle, as the images did not extend to the edge of the transducers) using a custom‐written Matlab script (Herbert et al. 2011, 2015; Diong et al. 2012, 2013) (Fig. 1). In the first frame of each image sequence, two fascicles were identified, and the deep and superficial aponeuroses were marked along with additional control points. The two clearest fascicles that could be visualized through the whole movement were then tracked through the image sequence. A fast normalized cross‐correlation algorithm with subpixel refinement was used to track displacements of control points across each frame (Herbert et al. 2011; Diong et al. 2012). After tracking the image sequence, linear regressions were fitted to the points marking the fascicles and cubic splines were fitted to the points marking the proximal and distal aponeuroses. The ends of the fascicles were defined as the intersections of the linear regressions with the cubic splines fitted to the aponeuroses. The distance along the tracked fascicles (proximal to distal ends) provided a measure of fascicle length (Herbert et al. 2015). The “effective” fascicle length (*l*_*f*_; the length of a muscle fascicle projected onto the long axis of the muscle) was calculated from the product of the measured fascicle length and the cosine of the pennation angle (Diong et al. 2012). All tracking was performed by one investigator.

For each muscle‐tendon unit, changes in whole muscle‐tendon length were estimated using change in joint angle data and published data on muscle moment arms at different joint angles. Equations relating change in moment arms with joint angle from Grieve et al. (1978) were used to calculate change in gastrocnemius length, equations from Klein et al. (1996) were used to calculate change in tibialis anterior length, and equations from Pigeon et al. (1996) were used to calculate change in brachialis length. Slopes (i.e., linear regression coefficients) of change in fascicle length on change in muscle‐tendon unit length were calculated using a “moving window” method: linear regression was performed over a window of samples comprising one‐third of the fascicle length and the regression coefficient determined. The window was moved to the next sample in time and the process repeated to determine the regression coefficient. In this way, regression coefficients were determined by shifting the window over the observed length of the fascicle. The maximum regression coefficient was used to indicate the relative contribution of the muscle fascicle to change in length of the muscle‐tendon unit. Pairwise comparisons of differences in median slopes between the three muscles and bootstrapped 95% CI were performed using R (version 3.3.0).

### Analysis of scaling

We hypothesized that the ratio of changes in muscle fascicle and tendon length scales linearly with the ratio of the slack lengths of the muscle fascicles and tendons. If that is true, a plot of the log of the ratio of changes in muscle fascicle and tendon length against the log of the ratio of the slack lengths of the muscle fascicles and tendons would be linear and would have a slope of 1 (Appendix 1).

To determine the ratio of the slack lengths of the muscle fascicles and tendon, we measured fascicle and tendon lengths from brachialis, tibialis anterior, and medial gastrocnemius muscles of five fixed cadavers. For each of these muscle‐tendon units, the muscle‐tendon length at the fixed joint angle was measured from the most proximal to most distal end of muscle. The tendon length was measured from the end of the most distal fascicle to the distal bone attachment. The fascicle length was taken from fascicle ends of the proximal and distal aponeuroses of tibialis anterior and gastrocnemius, or from ultrasound measurements of brachialis.

## Results

**Figure 2:**
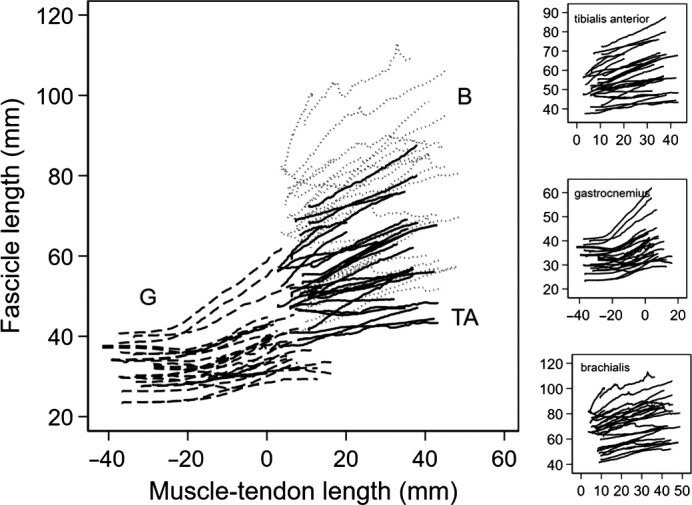
Individual participant data (two fascicles per participant) of change in fascicle length with change in muscle‐tendon unit length during passive lengthening. Data are shown for medial gastrocnemius (G: dashed lines), tibialis anterior (TA: solid lines), and brachialis (B: dotted lines). The main panel shows data for all three muscles and the smaller panels at right show the same data for each muscle. Muscle‐tendon length of 0 is nominally used to indicate ankle plantargrade and full elbow flexion.

Figure 2 shows changes in muscle fascicle length with change in muscle‐tendon length during passive lengthening, for two muscle fascicles from each muscle in all participants. The relationship between change in fascicle length and change in muscle‐tendon length was linear over the observed range of fascicle lengths of the tibialis anterior and brachialis muscles (*r* = 0.98 for tibialis anterior and 0.90 for brachialis). In the gastrocnemius muscle, the relationship was linear above slack length (*r* = 0.96).

In 3 of the 45 regressions of change in fascicle length on change in muscle‐tendon length, the regression coefficient was greater than 1. Regression coefficients greater than 1 are implausible because they imply that the fascicle lengthens more than the muscle‐tendon unit. It was assumed these values were overestimates of the true value. Consequently, they were assigned the maximum possible value of 1.

**Figure 3:**
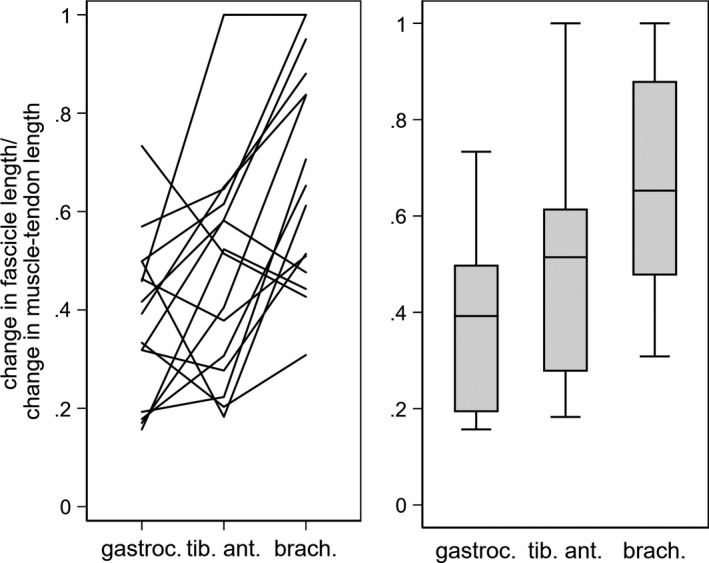
The ratios of change in fascicle length with change in muscle‐tendon unit length for the three muscles. Data are (left) individual raw data and (right) medians with interquartile ranges. G, medial gastrocnemius, TA, tibialis anterior, B, brachialis.

The median (IQR) contribution of the fascicle to lengthening of the muscle‐tendon unit, measured as the ratio of the change in fascicle length to change in overall muscle‐tendon unit length during passive lengthening, was 0.39 (0.26–0.48) in the medial gastrocnemius, 0.51 (0.29–0.60) in the tibialis anterior, and 0.65 (0.49–0.90) in the brachialis (Table 1). Brachialis muscle fascicles contributed to muscle‐tendon unit lengthening significantly more than those of medial gastrocnemius (difference of medians = 0.26, 95% CI 0.05–0.55, *P* = 0.01). However, the contribution by muscle fascicles to muscle‐tendon unit lengthening did not differ significantly between the tibialis anterior and medial gastrocnemius (0.12, 95% CI −0.12–0.26, *P* = 0.26), or the tibialis anterior and brachialis (0.14, 95% CI −0.07–0.53, *P* = 0.12; Fig. 3).

**Table 1:** Relationships between fascicle length and total muscle tendon length in three muscle tendon units

	Medial gastrocnemius	Tibialis anterior	Brachialis
*Δl*_f_/*Δl*_mtu_ observed in this study	0.39	0.51	0.65
*Δl*_f_/*Δl*_mtu_ reported previously	0.27 Herbert et al. (2002, 2011) 0.43–0.46 Herbert et al. (2002) 0.52^a^	0.55 Herbert et al. (2002)	‐
*Δl*_fs_/*Δl*_mtus_ observed in this study^a^	0.1	0.22	1.34
*Δl*_fs_/*Δl*_mtus_ reported previously	1:10 Herbert et al. (2002)	1:7 Herbert et al. (2002)	1:3 Murray et al. (2000) and Langenderfer et al. (2004)

mtu, muscle‐tendon unit.

a Observations on five cadavers.

**Figure 4:**
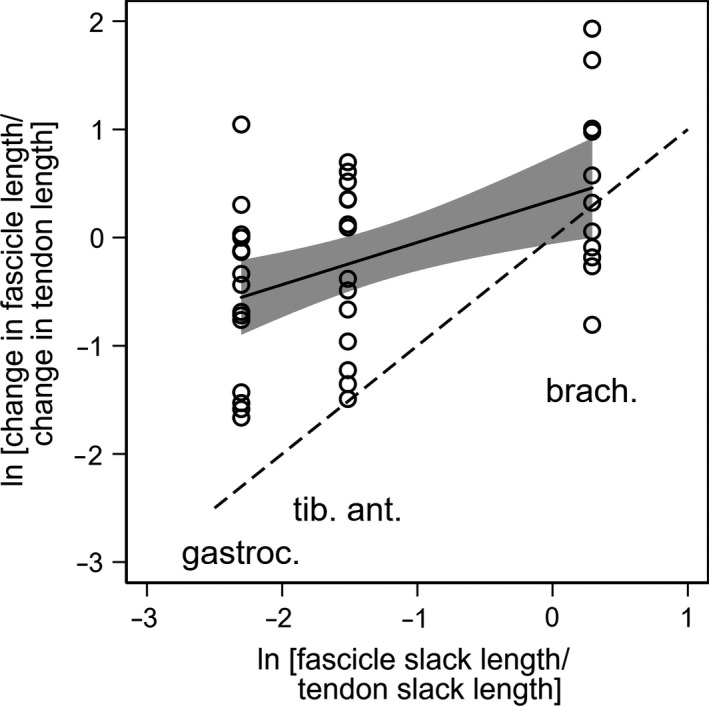
A plot of the log of the ratio of changes in fascicle and tendon length against the log of the ratio of muscle fascicle and tendon slack lengths. G, medial gastrocnemius, TA, tibialis anterior, B, brachialis.

The slope of the linear regression of the log ratio of change in muscle fascicle to tendon length against the log ratio of muscle fascicle to tendon slack lengths was 0.39 (95% CI 0.15–0.63; Fig. 4).

## Discussion

Brachialis muscle fascicles contributed more to passive lengthening of the muscle‐tendon unit (0.65) than fascicles in tibialis anterior (0.51) or medial gastrocnemius (0.39). Brachialis fascicles span approximately one‐third of the total muscle‐tendon unit length (Murray et al. 2000; Langenderfer et al. 2004), whereas medial gastrocnemius fascicles span approximately one‐tenth of the muscle‐tendon unit length (Herbert et al. 2002; Guilhem et al. 2016). While brachialis muscle fascicles contribute a greater proportion of the change in muscle‐tendon length than tibialis anterior and gastrocnemius, the contribution was less than might be expected on the basis of the relative slack lengths of the muscle fascicle and tendons alone (Fig. 4 and Appendix 1). This may be because the tendons of upper limb muscles are generally less compliant than the tendons of lower limb muscles. It has been hypothesized that this occurs because tendons of lower limb muscles need to be compliant to store elastic energy, whereas tendons of upper limb muscles need to be stiff to maximize fine motor control (Ward et al. 2006; Roberts 2016).

In some muscles, lengthening of muscle‐tendon units from very short lengths is not accompanied by any change in muscle fascicle length. This strongly suggests the muscle is slack (Herbert et al. 2011, 2015). We did not see evidence of slack in the brachialis or tibialis anterior, whereas we observed slack in the medial gastrocnemius for nearly all fascicles (Fig. 2). The finding of slack in the gastrocnemius muscle is consistent with the findings of our recent studies (Herbert et al. 2011, 2015). However, the finding that brachialis muscle fascicles lengthen over the full range of elbow motion contrasts with our claim, made in 1995, that brachialis fascicles are slack over most of the muscle‐tendon unit's physiological range of lengths (Herbert and Gandevia 1995). That claim was incorrect – the apparent lack of change in pennation with joint angle used to support that claim arose because the true change in pennation was small and could not be resolved with the methods available at the time which involved manual digitization of pennation (not fiber length) from low quality, static images generated by an early generation ultrasound system. By tracking fascicle length changes on video sequences of high‐quality images we have now shown that the brachialis muscle is not slack over most or all of its range.

The contribution by medial gastrocnemius fascicles to muscle‐tendon unit lengthening in this study (0.39) was larger than in some studies (0.27) (Herbert et al. 2002, 2011) but slightly smaller than calculated in others (0.43–0.46 (Herbert et al. 2002), or 0.52 at 60% of the in vivo range (Herbert et al. 2015); Table 1). The contribution by tibialis anterior fascicles to muscle‐tendon lengthening in this study (0.51) was similar to previous findings (0.55) (Herbert et al. 2002). There are no published data that can be used for comparison for the brachialis muscle‐tendon unit. Perhaps a combination of several methodological differences between studies may explain the minor observed differences in fascicle contributions to muscle‐tendon lengthening between studies. These include: (1) misalignment in the orientation of the ultrasound transducer may have occurred, which could result in potential errors measuring fascicle length and pennation angle (Bolsterlee et al. 2016); (2) differences in experimental set up (e.g., differences in knee joint angle); (3) differences in how fascicle lengths are defined (i.e., whether fascicle length was considered as the straight line distance between the insertions to the aponeuroses (Herbert et al. 2002), or as the length projected onto the long axis of the muscle (Herbert et al. 2015); (4) differences in methods used to estimate moment arms from joint angles; and (5) whether muscle slack lengths were included or excluded in analysis of final outcomes (Herbert et al. 2002, 2015; Hoang et al. 2005; Nordez et al. 2010). Given these many potential sources of variability in measurement, the differences between studies are surprisingly small.

All the participants in this study were healthy and recreationally active. However, their activity levels were not quantified; nor were sedentary, more elite sportspeople or older adults included in this study for comparison. Although tendon stiffness has been shown to alter with aging, disease conditions, and exercise training (Reeves 2006; Seynnes et al. 2009; Matschke et al. 2013; Grosset et al. 2014), we would expect that the contribution of fascicles to muscle‐tendon lengthening between distinct muscle‐tendon units in such populations would be similar to those observed in this study. However, further work in this area is warranted.

In conclusion, fascicles of muscle‐tendon units with relatively longer fascicles contribute more to passive lengthening of the muscle‐tendon unit than fascicles of muscle‐tendon units with relatively shorter fascicles. However, the contribution of fascicles to muscle‐tendon lengthening scales less than would be expected if the fascicle contribution scaled linearly with the ratio of muscle fascicle and tendon slack lengths.

## Conflict of Interest

The authors have no conflict of interest to declare.

## Appendix 1

Consider a muscle comprised of muscle fascicles in series with tendon. The muscle fascicles have length *l*_f_, slack length *l*_fs_, cross‐sectional area CSA_f_, and (under passive conditions, at lengths *l*_f_ > *l*_fs_) elastic modulus *K*_f_. The tendon has length *l*_t_, slack length *l*_ts_, cross‐sectional area CSA_t_, and (at lengths *l*_t_ > *l*_ts_) elastic modulus *K*_t_. The elastic modulus is likely to increase with muscle length so we write *K*_f_{*l*} and *K*_t_{*l*}.

With passive changes in muscle‐tendon unit length,

$$\frac{\Delta l_{f}}{\Delta l_{t}}=\frac{{\text{CSA}}_{t}}{{\text{CSA}}_{f}}\frac{K_{t}\left\{l\right\}}{K_{f}\left\{l\right\}}\frac{l_{\mathrm{fs}}}{l_{\mathrm{ts}}}$$

It might be reasonable to assume that the cross‐sectional areas of tendons are matched to the cross‐sectional areas of the muscles with which they arranged in series so that CSA_t_/CSA_f_ is constant across muscles. It might also be reasonable to assume that the material properties of muscle and tendon tissue vary little across skeletal muscles so that the ratio *K*_t_{*l*}/*K*_s_{*l*} is constant. Then

$$\frac{\Delta l_{f}}{\Delta l_{t}}=c\frac{l_{\mathrm{fs}}}{l_{\mathrm{ts}}}$$

where

$$c=\frac{{\text{CSA}}_{t}}{{\text{CSA}}_{f}}\;\frac{K_{t}\left\{l\right\}}{K_{f}\left\{l\right\}}$$

Taking logs:

$$\text{ln}\left[\frac{\Delta l_{f}}{\Delta l_{t}}\right]=\ln\left[c\right]+ln\left[\frac{l_{\mathrm{fs}}}{l_{\mathrm{ts}}}\right]$$

A plot of ln[*Δl*_f_/*Δl*_t_] against ln[*l*_fs_/*l*_ts_] is a straight line with intercept ln(*c*) and a slope of 1.
